# Modeling the seasonal wildfire cycle and its possible effects on the distribution of focal species in Kermanshah Province, western Iran

**DOI:** 10.1371/journal.pone.0312552

**Published:** 2024-10-28

**Authors:** Maryam Morovati, Peyman Karami

**Affiliations:** 1 Department of Environmental Sciences & Engineering, Faculty of Agriculture & Natural Resources, Ardakan University, Ardakan, Iran; 2 Water, Energy and Environment Research Institute, Ardakan University, Ardakan, Iran; 3 Department of Environmental Sciences, Faculty of Natural Resources and the Environment Sciences, Malayer University, Malayer, Iran; Government Degree College Totakan, PAKISTAN

## Abstract

Predicting environmental disturbances and evaluating their potential impacts on the habitats of various plant and animal species is a suitable strategy for guiding conservation efforts. Wildfires are a type of disturbance that can affect many aspects of an ecosystem and its species. Therefore, through the integration of spatial models and species distribution models (SDMs), we can make informed predictions of the occurrence of such phenomena and their potential impacts. This study focused on five focal species, namely, the brown bear (*Ursus arctos*), wild goat (*Capra aegagrus*), wild sheep (*Ovis orientalis*), wildcat (*Felis silvestris*), and striped hyena (*Hyaena hyaena*). This study used MODIS active fire data and ensemble machine learning methods to model the risk of wildfire occurrence in 2023 for spring, summer, and autumn separately. This study also investigated the suitability of habitats for focal species via SDMs. The predicted probability maps for wildfire risk and habitat suitability were converted to binary values via the true skill statistic (TSS) threshold. The overlap of the habitat suitability map and wildfire occurrence areas was analyzed via GAP analysis. The area prone to fire in spring, summer and winter is equal to 9077.32; 10,199.83 and 13,723.49 KM^2^ were calculated, which indicates an increase in wildfire risk. Proximity to roads is one of the most important factors affecting the possible effects of wildfires in all seasons. Most fire occurrences are concentrated on agricultural lands, which, when integrated with other land use types, have wildfire potential in all seasons. The use of fire to destroy agricultural residues is a critical factor in the occurrence of wildfires. The distribution range of each focal species is considered the most important component of fire susceptibility. Hence, the suitable habitat for *Hyaena hyaena* in spring, summer, and autumn, with areas of 5.257, 5.856, and 6.889 km^2^ respectively, is the most affected by the possibility of fire. In contrast, these areas have the lowest values for *Ovis orientalis*, with 162, 127, and 396 km^2^ respectively. Therefore, species that are dependent on human-based ecosystems have the highest vulnerability to wildfire. Conservation efforts should focus on familiarizing farmers with methods of destroying agricultural residues as well as the consequences of intentional fires. The findings of this study can be used to mitigate the negative impacts of wildfire and protect the habitat of focal species.

## 1. Introduction

Disturbance is a major concept in ecology [1] and has grown in importance over the past decade. Anthropogenic climate change currently presents a prime example of disturbance, which poses a considerable threat to nature, animals, and humans [2]. Climate change has various impacts, such as wildfires, that are becoming increasingly evident. Decreases in humidity and increases in heat periods as consequences of climate change increase the susceptibility of land to wildfire occurrence [3]. A warming climate will have profound effects on the extent, frequency, intensity and size of wildfires [4]. The impact of climate change on wildfires is so evident and significant that it is known as the main driver of the wildfire regime [5]. Since ancient times, fire has been a major force in human history and expansion, particularly at temperate latitudes [6]. Over time, the way in which humans use fire has changed considerably, and it is now used as a means to control the growth and development of nonbeneficial plants. In such circumstances, fire can be considered a useful tool, whereas if fire becomes uncontrollable, wildfire will occur as a widespread disturbance [7]. Wildfire behavior and the extent and frequency of occurrence can have various positive and negative effects on ecosystems. Controlling insects and diseases, producing seeds or seedbeds to restore vegetation, reducing competition between trees and increasing their growth, increasing biodiversity in some ecosystems, and expanding herbaceous vegetation are among the positive effects [8]. Wildfires affect the food chain of herbivores by destroying instantaneous food [9]. In addition, it strongly affects the physical, chemical, and biological contents of the soil and increases its erosion rate [10].

In ecology, complexity is a tremendous challenge [11]. Understanding complexity is gaining significance in light of accelerating global environmental change. Ecological systems exposed to multiple stressors frequently exhibit phenomena characteristic of complex systems [12]. Fires can effectively promote the sustainability of communities in diverse ecosystems. A common problem with sustainability is how to reduce dual ecological complexity to simple conditions [13]. The remotely sensed (RS) data can help assess wildfires in 3 circumstances: prefire, active fire, and postfire. The preparation of accurate maps of wildfire-affected areas for damage assessment, the development of effective strategies, and the analysis of the relationships among factors affecting the occurrence of fires are among the major types of data obtained from satellite images [14]. The major contribution of RS data is to provide different temporal–spatial scales for studies. The time scale can be monthly, yearly, seasonal, or long-term or short-term. Seasonal changes, akin to seasonality, are regular and periodic variations in eco-climatic conditions over the time scale of a year [15]. Therefore, RS data enable us to analyze wildfires in different stages and predict their effects. Seasonal remote sensing data can serve as a framework for mitigating ecological complexity. Land use change, disturbances (e.g., wildfires) and climate change affect the habitation of a wide range of wildlife species [16]. The impact of wildfire is so strong that it is regarded as a key threat to wildlife worldwide [17]. Wildfires alter and disrupt the wildlife habitats of many species by changing their vegetation structure [18, 19]; however, fires can also create new habitats for some species [20]. One of the fundamental ecological complexities is how fire can affect each species of an ecosystem. Many species are present in an ecosystem, and tools for assessing the impact of wildfire on biodiversity are very limited [21].

Spatial complexity has garnered significant attention because of its role in shaping patterns of vegetation and species distributions [11]. To reduce the complexity of the analysis, different definitions are considered by ecologists and conservation biologists. Thus, biodiversity conservation strategies often rely on the use of surrogate species [22]. One of the definitions proposed in this context is the definition of focal species by Lambeck (1997) [23], which makes different conservation strategies understandable to the public and stakeholders. These species can be selected on the basis of their sensitivity (e.g., the IUCN red list), response to landscape changes and future uncertainty components, and cultural, social, and environmental issues [13]. By selecting focal species and evaluating their distribution ranges, it is possible to estimate the effects of different environmental parameters on these species. Currently, SDMs are considered suitable solutions for assessing the susceptibility of plant and animal species and their habitats to changes [24]. In these models, the relationships between the presence of the species and the environmental variables are determined via algorithms, and finally, a suitability map is prepared. For example, Karami et al. (2023b) [25] used SDMs to investigate the effects of land surface temperature changes on the distribution range of *Salamandra infraimmaculata* in the Middle East. SDMs provide practical approaches for identifying suitable habitats for focal species [26, 27]. SDMs can determine the area and range of the environmental conditions of a focal species’ habitat, which facilitates the planning of conservation measures. A number of machine learning models, such as MaxEnt, can be used for wildfire modeling.

Understanding the susceptibility of a landscape to wildfire can help develop maps that depict varying levels of ecological vulnerability, hazard, and risk zones [28]. Currently, wildfire suppression is one of the major solutions for overcoming the problem of fire occurrence. If fire occurrence areas can be predicted via predictive models, the possible effects of fire on the habitat can be quantified. Machine learning models aim to create a set of guidelines or equations for estimating and mapping the variability in wildfire susceptibility across landscapes.

In many studies, RS data have been used to investigate wildfire probability, seasonal changes, and identify burned areas [29–31], which can subsequently be used to improve ecosystem conditions. Moreover, predictive models provide a robust framework for effective conservation planning. Wang et al. (2023) [32] investigated the effects of seasonal patterns and effective measures on fire occurrence via maximum entropy and random forest models in Yunnan Province, China. The findings revealed that areas prone to fire occurrence in spring and winter are scattered throughout Yunnan. During autumn and winter, fire occurrence areas are relatively concentrated. Differences in seasonal forest fire triggers reflect the delayed effects of climatic factors on these fires. Lewis et al. (2022) [7] studied the impact of fire plains on the habitat use of carnivores and large herbivores via remote wildlife (RW) cameras. It was predicted that the habitat use of animals would increase in heterogeneous areas suffering from fires of different severities (moderate, moderate/high, or high fire severity). In addition, road density can also have different impacts on habitat use. Some animals might increase their use near roads because of increased forage along roads or decrease their use because of increased human activity. Gao et al. (2023) [33] investigated forest fire risk via random forest (RF) and backpropagation neural networks (BPNNs) in the Heihe area in Heilongjiang Province, China. Forest fire data were collected from 1995–2015. Daily meteorological, topographic, and basic geographic data were also included in the modeling. RF and logistic stepwise regression methods were used to identify the most important drivers affecting the occurrence of fires. These results indicate that both models are suitable for predicting forest fire occurrence. The high-risk forest fire zones are concentrated in the northwestern and central parts of Heihe.

Kermanshah Province, in western Iran, covers various ecosystems. The northern parts are elevated and have plant and animal species specific to elevated regions, whereas the western parts have desert and hilly ecosystems. This ecosystem diversity has resulted in the presence of typical species of mountainous, forest, and desert areas, such as wild goats, wild sheep, brown bears, leopards (*Panthera pardus saxicolor*), roe deer (*Capreolus capreolus*), Kurdistan newts (*Neurergus derjugini*), Persian gazelles (*Gazella subgutturosa*), bustards (*Chlamydotis macqueenii*), spider-tailed horned vipers (*Pseudocerastes urarachnoides*), etc. In addition, Kermanshah has a special place in the agricultural sector of western Iran [34]. Studies on the fire trend showed that the incidence of fire in the forests and pastures of Kermashah during the study period had a slight but significant upward trend [35]. This special position, along with the dependence of the major part of Kermanshah’s economy on agricultural activities, has led to the use of fire as a tool to dispose of agricultural residues. In some cases, intentional fires by farmers lead to outbreaks of fires and their spread to protected areas. In addition to the loss of biodiversity and habitat destruction, it also leads to countless additional complications.

Temporal scales encompass monthly, seasonal, and annual intervals, as well as short-term and long-term durations. Seasonal variations, or seasonality, represent regular and periodic changes in environmental and climatic conditions over an annual timescale [25]. Examining seasonal environmental changes is a crucial component of ecological research that warrants significant consideration; however, this area has received limited attention. Various environmental factors influence the occurrence of wildfires, and these factors change dynamically with the seasons. Using RS data enables the study of phenomena across different temporal and spatial scales. Consequently, analyzing wildfire seasonality can provide deeper insights into the factors driving wildfires. The integration of RS data and machine learning predictive models can significantly enhance this research. To reduce the adverse effects of wildfires, accurate seasonal wildfire prediction is crucial for fire management and decision-making [36]. The objectives of this study are to: 1) assess the seasonal probability of wildfires, and 2) investigate the potential effects of wildfires on the distribution of focal species. This methodology allows researchers to analyze spatiotemporal fluctuations in fire-prone areas across different seasons and evaluate their potential impact on ecosystem biodiversity.

## 2. Materials and methods

### 2.1. Study area

**Fig 1:**
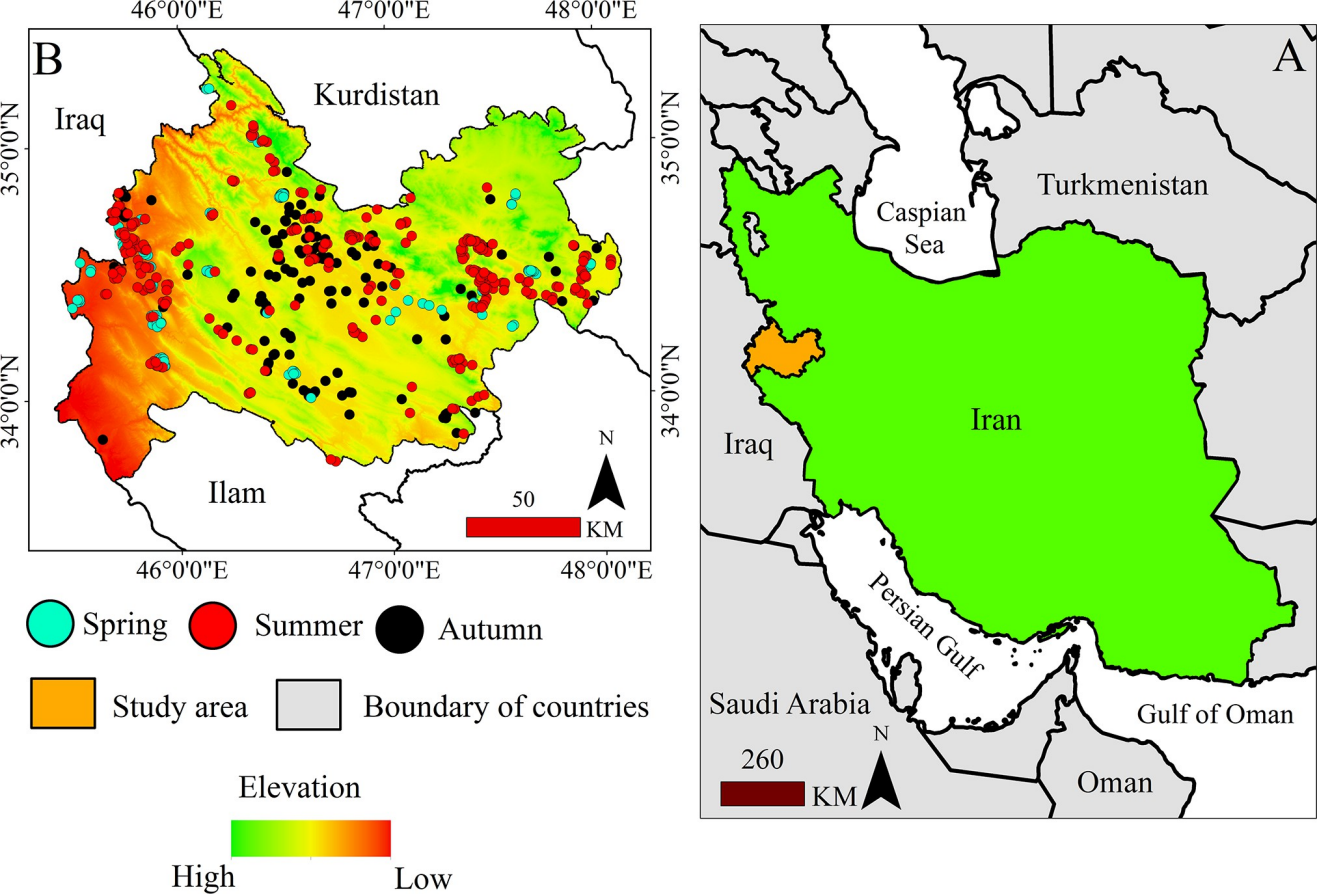
Location of the study area in western Iran (A) and the locations of fire occurrence points by season (spring, summer, autumn)(B).

Kermanshah Province, in western Iran, shares borders with Kurdistan Province to the north, Ilam Province to the south, Lorestan Province to the southeast, Hamadan Province to the east, and Iraq to the west. Fig 1 shows the location of the study area in Iran. This study focused specifically on the habitats of focal species within Kermanshah’s protected areas. However, as certain protected areas of neighboring provinces share a common border with Kermanshah, we also included the border of the protected areas shared between Kermanshah and neighboring regions in our analysis. Notable examples of these areas are the Shahu Kohsalan Protected Area adjacent to Kurdistan Province and the Qalajeh Protected Area adjacent to Ilam Province. Finally, the final border was established, as depicted in Fig 1.

### 2.2. Modeling the habitat of focal species

Different selection approaches have been employed to determine the focal species [37–39], which include *Ursus arctos*, *Capra aegagrus*, *Ovis orientalis*, *Felis silvestris*, and *Hyaena hyaena*. The selection was based on criteria such as body size, position in the food pyramid, and the number of presence points. The selected focal species are functionally diverse and include carnivores, herbivores, and omnivores. Field visits were conducted between 2022 and 2023 to record species’ positions via a global positioning system (GPS) and signs such as scat, skin, hair, and footprints.

We used the compound topographic index (CTI), digital elevation model (DEM), distance from the forest edge, distance from protected areas, distance from main roads, distance from springs, distance from residential areas, landscape diversity, density of mountain tops, heat load index (HLI), distance from poultry and cattle farms, average normalized difference vegetation index (NDVI) (2022–2023), normalized difference vegetation index (NDVI) roughness, slope, wind exposure index (WEI), escape terrain, and landform as variables. A digital elevation model (DEM) with a spatial resolution of 30 m was obtained from https://dwtkns.com/srtm30m/, and the CTI was computed from the digital elevation model (DEM) in SAGA GIS 7.8.2. The density of mountain tops was determined via Karami et al.’s (2023a) [40] method on the basis of landforms. The HLI was calculated from the DEM via the Geomorphometry and Gradient Metrics toolbox based on McCune and Keon’s method [41]. The northeast-facing slopes with a value of 0 were recognized as the coolest aspect, and those facing the southwest with a value of 1 were identified as the warmest aspect [42]. The habitat variable was also used for species with strong olfactory power, and it was prepared from the DEM via QGIS 3.34.2. The escape terrain is considered a crucial variable for wild sheep habitats [43]. The range of the escape terrain affects the behavior of a species when it uses other components of its habitat, as the activities of a species are often concentrated at a certain distance from it [44].

The land use map was derived from the map prepared by the Iranian Forests, Rangeland, and Watershed Management Organization, attained in 2010 from Landsat Enhanced Thematic Mapper Plus (ETM+) [45]. The map was used to determine the distance to the forest border variable. The land use/land cover map was first transferred to TerrSet to obtain the landscape diversity map. Using the Diversity H tool, the diversity of the landscape was subsequently calculated with a filter size of 5x5. The map of springs, protected areas, aviculture, and cattle breeding was also obtained from the Department of Environmental (DOE) of Kermanshah Province, and the Euclidean distance function was used to calculate the distance from these areas. Table 1 presents the environmental variables used in the modeling process and the range of values for each variable.

**Table 1:** Information on environmental variables used in the modeling process.

Number	Variable name	Minimum	Maximum	Source	Unit
1	DEM	113	3317	OpenTopography	Meter
2	CTI	3.20	17.69	DEM	-
3	Distance from forest edge	0	61774	Landcover	Meter
4	Distance from protected areas	0	51250	DOE	Meter
5	Distance from main roads	0	15789	DOE	Meter
6	Distance from springs	0	33547	DOE	Meter
7	Distance from residential areas	0	15967	DOE	Meter
8	Landscape diversity (H)	0.1	1.77	Landcover	-
9	Density of mountain tops	0	1	LandForm	-
10	HLI	0.3	1.12	DEM	-
11	Distance from poultry and cattle farms	0	28876	DOE	Meter
12	NDVI	-0.17	0.64	MODIS	-
13	NDVI roughness	0	0.99	NDVI	-
14	Slope	0.00	143.13	DEM	Percent_rise
15	WEI	0.82	1.34	DEM	-
16	Escape terrain	0	0.14	DEM	-
17	Landform	1	10	DEM	Class

Necessary preprocessing was performed on the presence points and variables. The distance between the wildfire presence points was calculated via Generate Near in ArcMap v.10.4.1. Presence points that were less than 1 km from the surrounding points were excluded from the analysis. A correlation test was also performed via BandCollectionStats, and variables with a correlation coefficient greater than 0.85 were removed from the analysis [46]. The habitats of these species were modeled via a generalized linear model (GLM), generalized additive model (GLM), multivariate adaptive regression splines (MARS), gradient-boosted trees, classification tree analysis (CTA), RF, artificial neural network (ANN), and support vector machine (SVM). All the maps were integrated on the basis of the AUC criterion [47], and the ensemble map was introduced as the final model. The mentioned models were run via the SSDM package [48]. Thirty percent of the data were used for testing, and 70% were used for training [40]. Then, using the TSS, the habitat suitability map was converted into a binary map. The detection threshold was evaluated via the AUC, sensitivity, and specificity criteria.

### 2.3. Preparation of wildfire occurrence data and variables

The wildfire occurrence data were prepared from MODIS active fire data at https://firms.modaps.eosdis.nasa.gov and from field visits in 2022 and 2023. Active fire data are MODIS sensor images based on the collection of 6 MODIS active fire detection algorithms, which are available to users [49]. The data were analyzed in spring, summer, and autumn. Winter data were not used in this study because, with the onset of rain and a decrease in temperature, wildfires occur with lower intensity in this season than in other seasons. Given that the proximity of wildfire occurrence points can increase spatial autocorrelation, the spatial resolution criterion was used to remove nearby points. Therefore, according to the spatial resolution of the data (300 × 300 m), points with distances of less than 300 m were excluded from the analysis [40]. Using wildfire occurrence data and a land-use/land-cover map, the percentage of wildfire occurrence by land-use/land-cover type was calculated. Wildfire occurrence is influenced by the complex relationships among climate, topography, vegetation cover, and human activities [50]. The effective variables affecting the occurrence of wildfires were identified on the basis of the parameters mentioned above and by reviewing the literature on the subject [51, 52]. The variables of DEM, distance from the road, seasonal average LST, seasonal average NDVI, landscape diversity, HLI, wind exposure index, and topographical openness were selected for the analysis. These variables have been cited the most in wildfire studies [53–55]. Dynamic variables have also been used to investigate wildfire risk as a method of investigating wildfire risk [56]. MODIS products were used to calculate the average LST and NDVI by season (MODIS/006/MYD11A1 and MODIS/006/MYD13A2 respectively). The spatial resolution was 250 m for the NDVI and 1 km for the LST. LST has greater spatial resolution than NDVI does; therefore, the average seasonal LST was downscaled by applying geographically weighted regression in QGIS. As wind can be effective in the spread of fire, the effect of wind was investigated using the wind exposition index, where values below 1 indicate wind-shadowed areas, and those above 1 indicate areas exposed to wind [57].

Topographical openness of the landscape refers to the relative position of valleys and heights. Instead of using multiple variables, this study used topographic openness, which reflects the extent of the landscape [58]. The output of the analysis was two maps that classified the land surface on the basis of concavities and convexities. After all the variables were prepared, the correlation between the variables was examined via a correlation test, on which variables with a correlation greater than 0.75 were excluded from the analysis. Table 2 presents the environmental variables used in the modeling process.

**Table 2:** Information on environmental variables used in the modeling process.

Number	Variable name	Minimum	Maximum	Source	Unit
1	DEM	113	3317	OpenTopography	Meter
2	Distance from the road	0	15789	DOE	Meter
3	HLI	0.3	1.12	DEM	-
4	Spring LST	289.59	322.96	MODIS	Kelvin
5	Summer LST	305.94	334.34	MODIS	Kelvin
6	Autmen LST	284.59	314.22	MODIS	Kelvin
7	Spring NDVI	-0.08	0.84	MODIS	-
8	Summer NDVI	-0.16	0.80	MODIS	-
9	Autmen NDVI	-0.14	0.69	MODIS	-
10	WEI	0.82	1.34	DEM	-
11	Negative Openness	0.52	1.77	DEM	-
12	Positive Openness	0.82	1.84	DEM	
13	Landscape diversity (H)	0.1	1.77	Landcover	-

### 2.4. Wildfire occurrence modeling

In this study, we predicted the probability of wildfires via classification models. Typically, input data for classification models should have at least two labels. However, owing to the lack of available data, we only had data related to wildfire presence. Thus, we prepared pseudo absence samples for wildfires via wildfire occurrence data. Models that employ this approach are commonly referred to as two-step pseudo absence selection methods. The method involves prior profiling of environmental data into classes [59]. After wildfire samples with spatial autocorrelation were removed, the remaining environmental variables were imported into the presence-only models of Bioclim, Domain, and one-class SVM, which model the probability of Wilfire via samples only. The models were implemented in ModEco [60], for which 70% of the data were considered for training and 30% for testing. The outputs of these models are binary. The AUC was subsequently used as a criterion for assessing the models [61]. Accordingly, models that have an AUC value greater than 0.8 are ensemble [42]. Following ensembling, a binary map is prepared with two values, 0 and 1, with 1 referring to areas of wildfire occurrence and 0 to regions with no wildfire risk. The zero-value areas were excluded from the final map, and the pseudo absence of wildfire events was considered twice the number of wildfire occurrences. The final dataset was entered into the Salford Predictive Modeler for modeling. Then, 70% of the data were considered for training, and 30% were considered for testing. The RF, CTA, MARS, TreeNET, generalized path seeker (GPS), and logistic regression models were used for modeling. As a review of wildfire studies confirmed the greater capability of the ensemble model [52], this study combined all the models on the basis of the AUC value to obtain the final map. The RF method was used to check the importance of the variables on wildfire occurrence [33, 61]. The TSS per season was used to convert the wildfire risk map into a binary map. Because temporal-spatial changes in wildfire risk are important for analyzing patterns, the probability map of each season was calculated via the following equation:

abs(b–a)/max(abs(b‐a))

where a and b refer to the wildfire risk in the previous and subsequent seasons, respectively.

## 3. Results

### 3.1. Wildfire analysis by land use/land cover type

The present study examines wildfire occurrence with respect to land use/land cover classes, as presented in Tables 3–5. As shown in Table 1, the majority of wildfires were recorded in urban peripheries (43.35%) during the spring season, while the lowest number of wildfires was reported in high-quality rangelands (0.05%). Notably, the summer season revealed a different pattern in terms of wildfire occurrence, with the integrated irrigated and rainfed agriculture as well as irrigated agricultural land use types experiencing the highest number of fires. A similar trend was observed in the autumn season.

**Table 3:** Frequencies of wildfire occurrence by different land use/land cover classes in spring.

Land use/land cover type	Frequency	Percentage
Agriculture	31	17.91
Rainfed agriculture	7	4.04
High-quality rangelands	1	0.05
Low-density forests	2	1.56
Integrated (rainfed and irrigated agriculture)	19	10.98
Integrated (rainfed agriculture and other land uses)	11	6.35
Semidense forests	3	1.73
Dense rangelands	10	5.78
Poor rangelands	14	8.09
Urban areas	75	43.35

**Table 4:** Frequency of wildfire occurrence by different classes of land use/land cover in the summer season.

Land use/land cover class	Frequency	Percentage
Agriculture	63	21.42
Orchards	1	0.34
Dense forests	3	1.02
Rainfed agriculture	12	4.08
High-quality rangelands	22	7.48
Low-density forests	2	0.68
Masil(Streams, watercourse)	3	1.02
Integrated agricultural and orchard land uses	19	6.46
Integrated irrigated and rainfed agricultural land uses	82	27.89
Integrated rainfed agriculture and other land uses	23	7.82
Integrated poor rangelands and other land uses	2	0.68
Integrated low-density forests and other land uses	2	0.68
Semidense forests	15	5.1
Semidense rangelands	12	4.08
Poor rangelands	23	7.82
Urban areas	9	3.06
Very low-density forests	1	0.34

**Table 5:** Frequency of wildfire occurrence by different classes of land use/land cover in the autumn season.

Land use/land cover class	Frequency	Percentage
Agriculture	14	10.60
Rainfed agriculture	2	1.51
High-quality rangelands	1	0.75
Low-density forests	3	2.27
Integrated agricultural and orchard land uses	2	1.51
Integrated irrigated and rainfed agricultural land uses	59	44.69
Integrated rainfed agriculture and other land uses	32	4.24
Integrated low-density forests and other land uses	1	0.75
Integrated semidense forests and other land uses	1	0.75
Integrated semidense rangelands and other land uses	5	3.78
Integrated poor rangelands and other land uses	2	1.51
Integrated very low-density forests and other land uses	1	0.75
Semidense forests	3	3.27
Semidense rangelands	2	1.51
Poor rangelands	4	3.03

### 3.2. Wildfire risk

Table 6 shows the validation results of the different wildfire occurrence models by season. The computed AUCs for different seasons are high, indicating the reasonable efficacy of the individual models. The sensitivity values in the spring season surpass those in the other seasons, suggesting that the model accurately identifies wildfire occurrences in this season. The model has a specificity value of 93.4% for the spring season. Conversely, the model’s detection power for wildfire occurrence in the summer season is lower than that in the spring season, and the sensitivity value decreases by 74%.

**Table 6:** Assessment of the ensemble models used in this study.

Season	AUC	Threshold	Sensitivity	Specificity	Correct classification	Misclassification
Spring	0.97	0.54	93.4	93.4	0.93	0.066
Summer	0.91	0.52	74	93	0.83	0.16
Autumn	0.93	0.44	87.9	86.4	0.87	0.12

**Fig 2:**
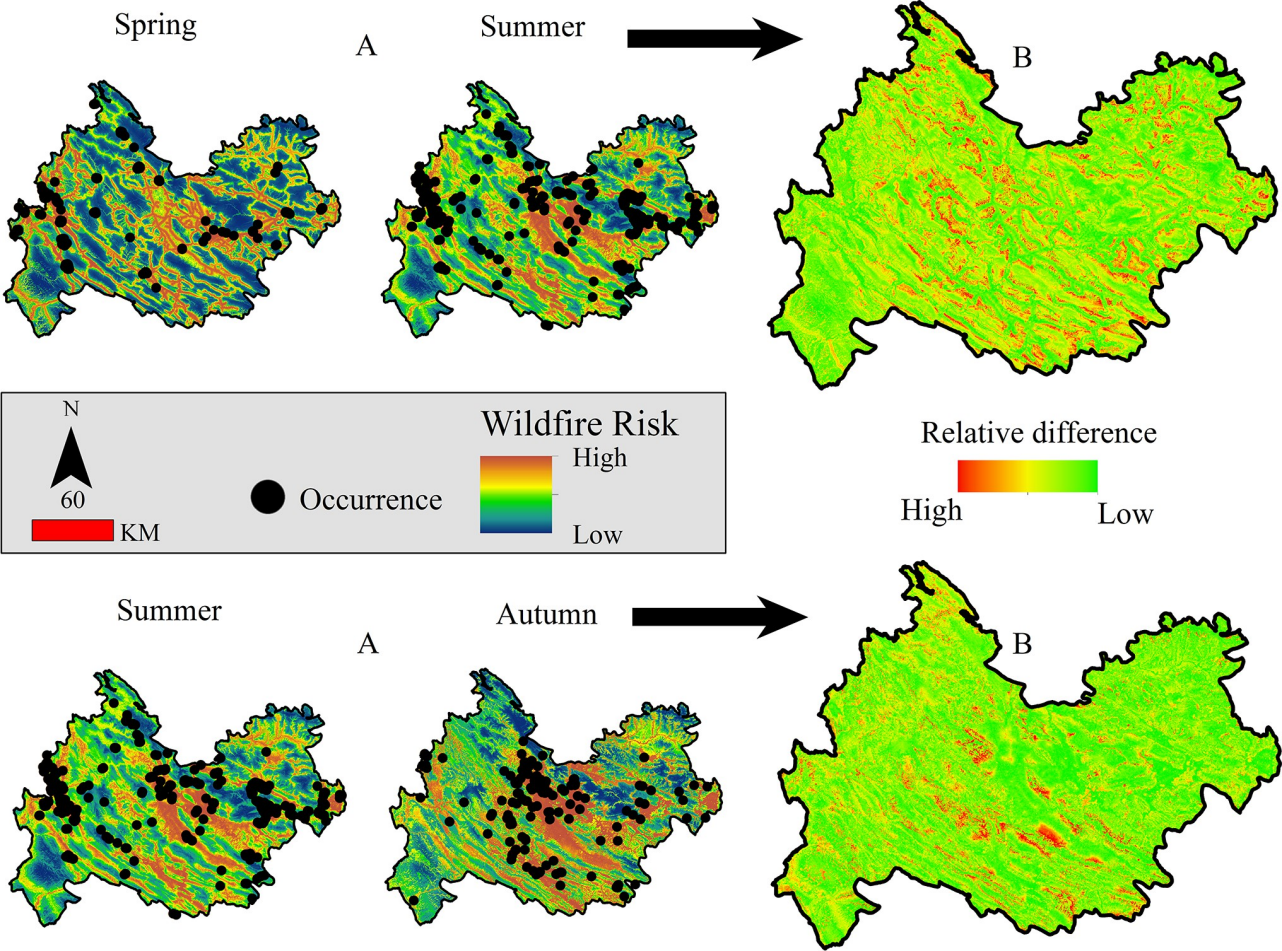
Wildfire risk (A) and its fluctuations across the study area in the spring, summer, and autumn seasons (B).

Fig 2 shows the areas susceptible to wildfires during the spring, summer, and autumn seasons. The regions at high risk are depicted in brown, while low-risk territories are displayed in blue, and the black dots indicate instances of wildfire. In the spring season, areas prone to wildfire risk have a linear arrangement. The two large maps on the right side of the figure offer a relative comparison of wildfire risk for the two seasons. The green color on these maps denotes a low difference in wildfire risk between the seasons, whereas the red color implies a significant difference. The summer season experiences an increased range of wildfire risk, covering a larger expanse of roadways. The range of wildfire risk is further exacerbated during autumn.

**Fig 3:**
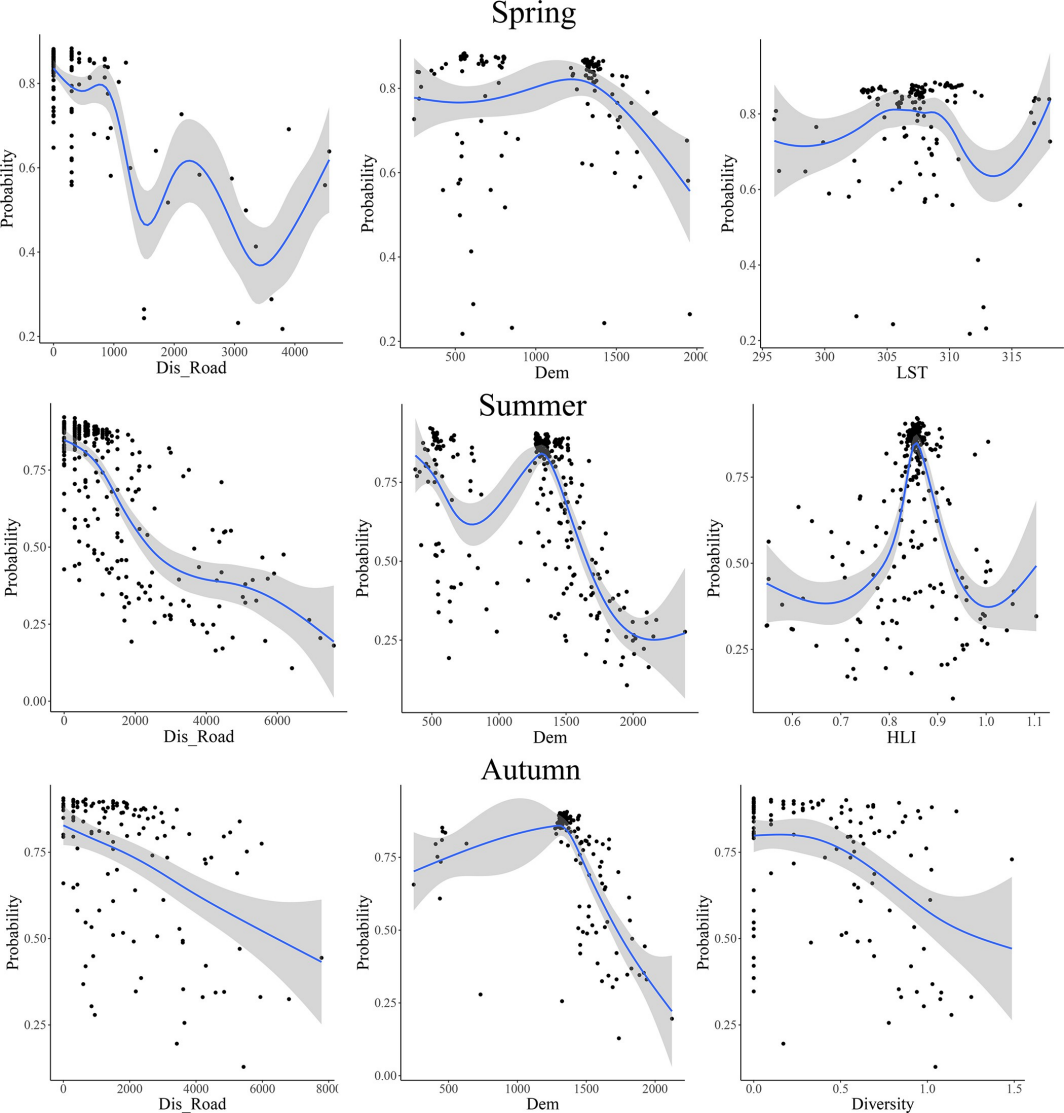
Response curves of the variables affecting wildfire risk by season.

Table 7 shows the variables influencing wildfire risk in different seasons, namely, spring, summer, and autumn, according to the RF method. The table highlights that elevation, distance from the road, and average LST are the most influential factors during the spring season. In summer, elevation and distance from the road are the most significant variables affecting HLI. During the autumn season, the DEM, distance from the road, and landscape diversity had the most significant impacts on wildfire risk. The results indicate that distance from the road and elevation are key factors in all three seasons. The response curves of the three important variables in each season, along with the wildfire probability, are illustrated in Fig 3. The Figur reveals that the risk of fire in the spring season decreases as the distance from the road increases. Additionally, the wildfire risk increases with increasing altitude up to 1500 m and then falls beyond that altitude.

**Table 7:** Weights of influencing variables on modeling by season.

		Weight	Autumn
Variable	Spring	Summer	Autumn
DEM	0.19	0.19	0.20
Dis-Road	0.16	0.14	012
Mean LST	0.1	0.1	0.11
Negative Openness	0.09	0.08	0.08
Diversity	0.09	0.11	0.11
Mean NDVI	0.08	0.08	0.11
HLI	0.08	0.119	0.1
Positive Openness	0.08	0.09	0.06
Wind exposition	0.08	0.06	0.06

### 3.3. Habitat suitability and wildfire risk

The results obtained from the validation of the SDMs are summarized in Table 8. The AUC values indicate that the models exhibited good performance overall. In particular, the highest AUC score was obtained for the habitat of the wild sheep. The TSS value for the corresponding probability map was 0.30. At the threshold for this map, the sensitivity and specificity values were 0.93 and 0.98, respectively. These results are considered acceptable within the context of the modeling conditions.

**Table 8:** Validation of the SDMs by focal species.

Species name	AUC	TSS	Sensitivity	Specificity
*Capra aegagrus*	0.95	0.21	0.87	0.93
*Ovis orientalis*	0.97	0.30	0.93	0.98
*Ursus arctos*	0.93	0.22	0.87	0.91
*Hyaena hyaena*	0.84	0.09	0.77	0.76
*Felis silvestris*	0.84	0.30	0.88	0.76

**Fig 4:**
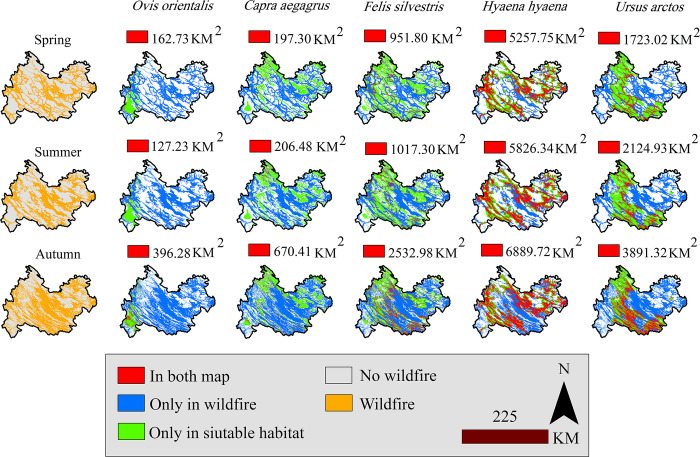
Overlap between the suitable habitat of focal species and wildfire risk.

Fig 4 highlights the potential overlap between the habitats suitable for the focal species and the areas at risk of wildfires. The figure presents three instances where the suitable habitats of the focal species and the wildfire-prone regions may overlap. The red areas indicate that the habitats of the focal species are at risk of wildfires. The figure shows that the habitat of hyenas, which spans an area of 5257.75 km^2^, is the most vulnerable to wildfires in the spring season. The brown bear habitat, covering an area of 1723.02 km^2^, ranks second in this list. Blue indicates areas that are at risk of wildfire but are not suitable habitats. Green refers to areas that are suitable habitats but not at risk of wildfire.

## 4. Discussion

This study endeavors to analyze the impact of wildfires on the habitat suitability of focal species in a seasonal context via machine learning approaches. The results revealed that the ensemble models were efficient at discriminating between wildfire-affected areas and nonaffected areas. During the spring season, the sensitivity was estimated to be 93.4, and the specificity was 93.4, which falls within the acceptable range. However, during the summer season, the sensitivity was rather low, and only 74% of the wildfire-affected areas were identified successfully. Notably, the specificity for areas where wildfires do not occur was 93. The reason for the low specificity in summer may be the high complexity of the environment. Reliable estimation of wildfire susceptibility is challenging because of the intricate interplay of geological, topographical, and environmental factors across the landscape [28].

The occurrence patterns of wildfires vary by land use/land cover in different seasons, particularly in spring. Certain regions, such as forests, rangelands, and agricultural lands, which are dependent on the density of land cover, are more susceptible to wildfire occurrence in specific seasons than others are, which indicates that not all land use/land cover types are equally at risk. Over the past few decades, land use/land cover changes and the development of agricultural lands through clear-cutting forests, indiscriminate livestock grazing, and the presence of nomadic herders have been among the major concerns that have led to changes in vegetation cover and land use in the study area. These changes have the potential to disturb the microclimate. Changes in microclimatic conditions, such as increasing temperatures, have led to the loss of some structural aspects of habitats [62]. A number of studies have also acknowledged that some areas may not be prone to fires. Ashton and Zhu (2020) [63] revealed that evergreen, semievergreen, and montane temperate forest types have the lowest probability of wildfire occurrence.

Among the environmental factors affecting wildfires, roads play a crucial role in all seasons. The existence of roads means greater accessibility. The discarded flammable materials (such as water bottles or cigarette filters) on roads can act as fuel to start a fire. Human carelessness, particularly on the part of drivers, herders, and hunters, is one of the major factors contributing to fires in the forests of Iran [64–66]. Numerous studies h ave also shown that many wildfires occur near roads [67–70]. The role of proximity to roads is more significant in spring than in autumn and winter. Roads can also act as barriers to wildfire propagation [68]. The intensity of human-caused wildfires is closely influenced by road density and distance from roads. In all three seasons, elevation was the second most important factor in the occurrence of wildfires. The influence of topographical factors on wildfires has been confirmed in previous studies [71]. However, the severity of wildfire impacts in an area can also depend on other environmental parameters, such as land use/land cover. In spring, wildfire events at higher elevations usually occur at elevations of up to 1500 m, whereas in summer and autumn, the elevation increases, reaching 2000 m. Therefore, the extent of wildfire occurrence increases from spring to summer and from summer to autumn, which is in line with the results of a study by Balch et al. (2017) [72]. The occurrence of wildfires in highlands and forests is associated with elevation changes. In a study by Ghanbari Motlagh et al. (2022) [73] on Zagros Oak forests, the elevation of 500–1500 m had the highest probability of fire occurrence, which aligns with the findings of the present study.

The seasonal cycle of changes in vegetation cover and temperature can be among the major factors influencing the occurrence and increasing risk of wildfires [74]. In tropical regions west of the study area (Fig 1), which are characterized by low altitudes and high temperatures, agricultural products are harvested faster, leading to reduced humidity and increased dryness. This explains why the occurrence of wildfires is greater in these areas in spring and summer but lower in autumn (Fig 4). A study conducted by Charizanos and Demirhan (2023) [75] revealed that dry and hot conditions can increase the potential for wildfire occurrence.

Numerous studies suggest that a relationship exists between wildfires and topographic factors (such as elevation and slope), such as Rodriguez-Jimenez et al. (2023) [76]. However, it should be noted that the impact of these factors can vary based on the specific geographical region, as indicated by Ríos-Pena et al. (2017) [77]. Understanding these dynamics is crucial for informed wildfire management and future ecological restoration efforts. Elevation was the dominant variable influencing wildfires, followed by a mean LST between 300 and 315 K, which was the second most influential factor for spring fires. In summer, HLI serves as the third major variable. The exposure to higher solar radiation, the extreme summer heat in 2023 and the loss of moisture at higher altitudes with strong wind probabilities can cause wildfires in summer. The prevalence of forest fires in recent years due to land dryness is also noteworthy. When vegetation is harvested, the soil is exposed to greater amounts of solar radiation. Heisler et al. (2004) [78] confirmed that the loss of vegetation cover can lead to increased soil temperature. In addition, wildfires, as geophysical phenomena, have also increased in frequency as a result of global warming [56]. The third influential factor in the autumn season is the variety of land use. As the variety of land use types increases, the risk of wildfire decreases. This confirms that certain land use/land cover types are more prone to wildfires.

The deliberate use of fire to manage agricultural waste, aka crop residue burning or stubble burning, is a fast and cheap method to clear crop straw. In this case, fire can be proposed as a tool for the easy and quick removal of plant residues in agricultural fields. Wildfires in summer and autumn are due to fuel availability, which plays an important role in the occurrence of these types of fires [79]. Several studies have revealed that lightning is an important factor in the occurrence of wildfires [55]. However, in this study, the high incidence of wildfire in the combined irrigated/rainfed agricultural lands and irrigated agricultural lands can be due primarily to intentional fires to remove crop straw and the possibility of its spread by the wind. This leads to uncontrollable wildfires, which often spread quickly across nearby rangelands and forests. In addition, the water and nutrients absorbed from the soil during cultivation are wasted; that is why the combination of agriculture with other land use/land cover types makes the land more prone to wildfire events (Tables 3–5).

However, Kermanshah Province is the hub of agricultural production in western Iran, with the majority of its cities being dependent on agriculture for their livelihoods [80]. The same is true for Golestan Province in northern Iran [69]. Wildfire occurrence increased with proximity to urban areas, which highlights the role of intentional fires. This has also been reported in other studies [81]. Fire mitigation measures should focus on the proper disposal of agricultural biomass. Straw mulch is one of the most affordable and sustainable organic-based management practices among farmers. Reports show that straw mulch can store water in the soil [82], protect the soil from raindrops [83], reduce erosion and sedimentation [84], increase the roughness of the soil surface, reduce the volume and speed of surface flow, increase the content of soil organic matter, strengthen the soil structure [85], and improve the capacity of water penetration into the soil [86].

Spatial extent, intensity, and frequency are important characteristics of disturbance [87, 88]. Different plant and animal species are affected by fire in different ways. Plants have acquired various adaptations against fire. SDMs represent a powerful tool for biodiversity assessment and conservation. Given the vast area of the habitat, the variety of temporal scales, and the diversity and vastness of biodiversity, if we aim to assess the effects of environmental changes on biodiversity, the use of SDMS for focal species is an effective approach. Lõhmus et al. (2020) [13] argued that the concept of focal species conservation links multiple ecological issues that can be addressed via habitat modeling.

The present study revealed that a vast portion of the forests in the study area are exposed to fires in different seasons (Tables 3–5). In these forests, shrubs and bushes are more likely to burn more severely than taller and less flammable trees are. However, plant species have acquired certain adaptations against fire, including resprouting. In the studied forests, the resprouting process was observed for a number of species, such as *Quercus brantii*, *Quercus infectoria*, *Prunus microcarpa*, *Amygdalus lycioides*, and *Acer monspessulanum*, but not *Pistacia atlantica* [89]. Coppice regeneration makes oak forests susceptible to fire. Species dispersal ability can be a determining factor in the recovery patterns of plant species following a wildfire event [90]. Owing to the low susceptibility of Zagros forests to spring fires, the species dependent on these forests were less affected by fires in this season. In other seasons, an increase in fire occurrence was observed.

Fires can impact large carnivores by affecting both the shelter and habitat quality of their prey [91]. The effects of fire on focal species can be strongly influenced by the spread and range of the species as well as the intensity and frequency of the fire [20]. In a fire with large spatial dimensions, large species are usually more likely to be damaged because of the greater impact on their habitat. For species that are dependent on mature forests, there is a direct negative relationship between the magnitude and intensity of fires and habitat quality [92]. With an increase in habitat vulnerability and the loss of resources such as food and shelter, species may move closer to residential areas [93], leading to increased conflict between wildlife and human communities [94]. The brown bear is a prominent example of human‒wildlife conflicts in this region, with numerous reports of bears venturing into orchards adjacent to protected areas and agricultural lands near dense oak forests. Similarly, Ziółkowska et al. (2016) [95] reported that brown bears of the Carpathians favor areas with dense forest cover close to forest edges under low human pressure. Therefore, it could be argued that human‒wildlife conflict can be associated with an increase in wildfire intensity. Brown bears, which are widely regarded as umbrella species [96], can play crucial roles in the survival and distribution of plant species. Wildfires can change animal‒plant interactions by affecting the endozoochory dispersal mechanism of seed dispersers such as the brown bear [97]. The roe deer, a species commonly found in the brown bear’s habitat, will also be at great risk with the loss of dense forest cover. In a study by Cherry et al. (2017) [98] on *Odocoileus virginianus*, the results revealed that the species tends to disappear from burned areas following wildfires. However, given the proximity of the roe deer distribution area to the Iraqi Kurdistan region, temporary migration is a possible postfire strategy. Temporary migration is a useful coping strategy for species with high mobility, such as the brown bear and the roe deer, but not for small species, such as the Persian squirrel (*Sciurus anomalus*). This species depends on oak, olive, and broad-leaved trees to create cavities for nesting [99]. Therefore, among the species that fall under the brown bear conservation umbrella, small species are the most susceptible to wildfires.

The dependence of the striped hyena on forest habitats and low- and high-elevation hills has led to its high susceptibility to wildfire. The broad ecological niche of this species, as documented in previous studies [100], has resulted in its habitat overlapping with several small mammal species [101]. The striped hyena often ventures into agricultural fields, villages, and residential areas at night to avoid herd dogs and humans. The vulnerability of the habitat of this species suggests habitat vulnerability of many other animal species that may occur in human-modified environments, such as the long-legged buzzard (*Buteo rufinus*), wolf (*Canis lupus*), red fox (*Vulpes vulpes*), lesser kestrel (*Falco naumanni*), and common kestrel (*Falco tinnunculus*) observed in this study. The low degree of habitat overlap with wildfire risk areas in summer may be due to agricultural and animal husbandry activities during this season. Similar results were reported by Bhandari et al. (2021) [102] in a study in a striped hyena habitat in Nepal. After brown bears and striped hyenas, wildcats suffer the most from wildfires. The species exhibits ambiguous responses to wildfires. Studies have revealed that feral cats (*Felis catus*) in northern Australia are drawn to burned areas with populations of small mammals [103]. Owing to changes in the habitat structure of prey species, the success of hunting for the wildcat is expected to increase, which will lead to the presence of the species in burned areas. In a study by Pastro (2013) [104] on feral cats in Australia, it was found that cats’ preference for burned areas was greater in ecotones than in other areas. However, some studies report reduced activity in cats following a fire [105, 106]. Therefore, further research is needed to investigate how carnivores respond to fire.

The vulnerability of ungulates to wildfires depends on the availability of fuel for fires at high altitudes. Wild goats are more affected by fire than are wild sheep. The habitat of wild goats partly overlaps with the Zagros forest cover, making the species susceptible to habitat loss in areas of overlap. Moreover, wild sheep are often distributed in the eastern hills of Kermanshah, which are less vulnerable to fire. The wild goat, as a browsing species [107], can suffer from far greater consequences following changes in forest canopy cover. Fire can affect the quantity and quality of forage available to wild goats [108]. As ungulates normally have a clustered distribution and occur mainly in highlands, they can be less affected by fire than the other species in this study. However, this small effect appears to have a more severe impact on their habitat. Studies have shown that in areas with lower fire intensities, vegetation cover recovers to prefire conditions, both in terms of quantity and quality, after one to three years [109].

## 5. Conclusion

Analyzing wildfire dynamics and predicting their occurrences, when combined with forecasts, can effectively mitigate potential negative impacts. Seasonal wildfire analysis is a crucial tool for understanding wildfire dynamics in the landscape. Future studies should focus on wildfire forecasting, emphasizing seasonal and monthly time scales, and using land use/cover maps to make wildfire dynamics more apparent.

Altitude is an important factor in the pattern of fires in Kermanshah. This factor can distinguish the occurrence of fires in the western tropical regions of the province in spring and summer from the eastern regions. Therefore, fire management programs can be carried out according to the season and with emphasis on the height classes identified in this study. forecasts indicate a rising probability of wildfires from spring to autumn. This increase is partly due to the use of fire for destroying agricultural crops. It is crucial to educate farmers on biologically and ecologically sustainable methods for crop straw disposal. Priority should be given to farmers working near forested areas. The role of the road is also very important due to facilitating access to fire-prone lands in hot and dry seasons, so in this study, proximity to the road is considered as an important factor in the 3 seasons examined. Providing brochures and educational programs to road users in hot and dry seasons, especially in summer, in order to inform them of the dangers of ignition and leaving plastic can be effective measures.

This study underscores the vulnerability of wildlife species dependent on human ecosystems to wildfire incidents. The habitat assessment of the focal species indicates that wildlife species that depend on human ecosystems are more vulnerable to fire than other species are. The risk of vulnerability decreases with increasing distance from infrastructure and human settlements. Hyenas, brown bears, and wildcats are among the most susceptible to fire incidents. Using the findings of this study, it is possible to identify areas with high wildfire risk and take appropriate action to reduce its adverse environmental effects. To mitigate the impact of wildfire on focal species, land managers can implement various strategies, including mechanical thinning, creating firebreaks, and conducting controlled burns. These practices help reduce fuel loads and create more resilient ecosystems.

This study offers valuable insights into the factors influencing wildfire occurrence and identifying high-risk areas. Additionally, an important parameter affecting wildfire intensity is the landscape’s connectivity. Awareness of the spatial connectivity of wildfires provides useful information for wildfire hazard and fuel management plans. Studying the spread of fire and its landscape connectivity can be the next step to complete the results of this study.

## Supporting information

S1 File https://figshare.com/articles/dataset/_b_Modeling_the_seasonal_wildfire_cycle_and_its_possible_effects_on_the_distribution_of_focal_species_in_Kermanshah_Province_b_b_western_b_b_Iran_b_/26879107. (PDF)
