# Supplementary material for: Modeling the seasonal wildfire cycle and its possible effects on the distribution of focal species in Kermanshah Province, western Iran
Source: PLoS One. 2024 Oct 28;19(10):e0312552. doi: 10.1371/journal.pone.0312552 (PMC11516172; doi:10.1371/journal.pone.0312552)
Supplement: S1 File — (PDF) [file pone.0312552.s001.pdf]

-Where did the authors obtain the maps, basemaps, shapefiles, map data, etc in Figure 1?

In this version, we have indicated the address to receive all the necessary information for the implementation of this study

In the initial version of the article, the google earth base map was used in Figure 1. But in the revised version, this figure was removed.

Figure 1 in the revised version includes the following sections
1- Map of world countries (A)
2- Map of different provinces of Iran (B)
3- Altitude map masked to the border of the study province (C)
4- And map of seasonal fires (D)
Below are the addresses through which the shape files can be received

A -The global shape file of the borders of the countries can be obtained from different addresses
- https://diva-gis.org/
- https://public.opendatasoft.com/explore/dataset/world-administrative-boundaries/export/
In this study, address (https://diva-gis.org/) was used. In this site, there are various sections including software and data. Currently the site under reconstruction.

B- The second map was prepared by the Ministry of Iran. This map is freely available in most departments and organizations that deal with natural resource sciences and agriculture.
This map can be easily downloaded from the following addresses

https://data.humdata.org/dataset/cod-ab-irn?

In this site, the second item with the name (irn_adm_unhcr_20190514_SHP) provides all the information used about the different provinces in Figure 1.

C- Inside the method, it is also mentioned that the height is received from the following address
https://dwtkns.com/srtm30m/

D- The source of fire points is also mentioned in the method. These points can be obtained from the following address
https://firms.modaps.eosdis.nasa.gov/country/"
